# Evaluating the Mechanism by Which the TNO Stereo Test Overestimates Stereo Thresholds

**DOI:** 10.1155/2021/6665638

**Published:** 2021-01-18

**Authors:** Yu Zhang, Bingbing Meng, Huang Wu

**Affiliations:** Department of Optometry, Second Hospital of Jilin University, Changchun, China

## Abstract

Several studies have revealed that results of the TNO stereo test may overestimate the stereoacuity value (the less the better) compared with other testing measurements. The manner in which vision is divided among two eyes of a person wearing anaglyph glasses may play an important role. This study aimed to examine the effect of anaglyph glasses on stereopsis measurements. A stereopsis measurement system using a phoropter and two Sony smartphones was established. Four types of test patterns, including the original TNO stereo test pictures, isoluminant red-green pictures, grayscale pictures, and black and white dots pictures, were designed. A total of 32 participants were recruited for this study. A significant difference was found among the four groups (Friedman test, chi-square = 50.985, *P* < 0.001). The Wilcoxon signed-rank test was used to detect differences between the groups. The stereoacuity of the original TNO group was significantly worse than those of the isoluminant, grayscale, and black-white groups. However, no significant difference was found between the isoluminant and grayscale groups. The correlation coefficient between the original TNO and isoluminant groups was 0.952 (Spearman's rho, *P* < 0.001; 95% confidence interval (CI), 0.901–0.988), while that between the original and grayscale groups was 0.771 (Spearman's rho, *P* < 0.001; 95% CI, 0.550–0.916). Anaglyph glasses played an important role in determining the stereoacuity values with the TNO stereo test, and the results were overestimated when compared with that of the other testing methods. The imbalance of chroma and luminance between the two eyes caused by the anaglyph glasses was indicated as one of the reasons for the overestimation of stereo thresholds.

## 1. Introduction

Stereopsis is a kind of binocular function which helps to judge distance precisely. Some disorders involving binocular, such as amblyopia, not only affect visual acuity but also impair stereopsis [1, 2]. The TNO stereo test is a commonly used clinical test to evaluate stereopsis and should be conducted while wearing anaglyph glasses. The test pattern was designed with a random dot, and the latest version, 19^th^ edition, contains six test plates. Several studies have revealed that the test results of the TNO stereo test may overestimate the stereoacuity value (the less the better) compared with other test measurements in populations with either normal or abnormal binocular functions [3–10]. The possible mechanism has been discussed for several decades. The manner in which the two eyes are divided when using the anaglyph glasses may play an important role. Red and green filters placed in front of the left and right eyes, respectively, can lead to at least two differences between the two eyes. The first difference is chroma. The right eye observes the test materials through a green filter and they appear green, while the left eye watches the test pages through a red filter and the materials appear red. The two images with different colors should be fused together to obtain stereopsis, which may lead to a higher result than the actual threshold. The second difference is the grayscale of the test images. In our previous study, the transmittance of the red filter was higher than that of the green filter [11]. That is, the image seen by the right eye with a green filter was darker than that of the left eye with a red filter. The difference in luminance between the two eyes may also interfere with stereopsis. It was difficult to determine the exact thresholds affecting stereopsis measurement with chromatic or luminance differences. However, it was possible to evaluate whether the chromatic or luminance differences between the red and green filters of the TNO stereo test itself may affect the test results [11].

In the quantitative test of the TNO stereo test, the minimum disparity setting is 60 seconds of arc (arcsec, ″), and the step ranges are 60″, 120″, and 240″. It is difficult to obtain a reliable comparison of stereopsis with other tests that use different step ranges. We designed a test system with two 4K resolution smartphones and a phoropter that could achieve a minimum disparity setting of 10″ and maintain step range increments of 10″. A series parameter was obtained with TNO red-green glasses, and simulation models were set up to evaluate the effect of stereopsis with different chroma and luminance between the two eyes.

## 2. Materials and Methods

### 2.1. Test System

**Figure 1:**
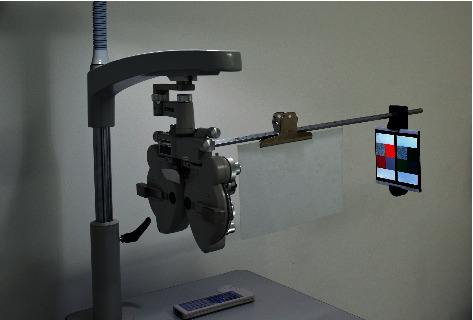
Photograph of the actual test.

A stereopsis measurement system using a phoropter (Topcon VT-10, Topcon Corp, Tokyo, Japan) and two Sony smartphones (Sony Xperia Z5 Premium Dual E6883; Sony Mobile Communications Inc., Tokyo, Japan) was established [12, 13]. The test distance was set at 65 cm at which a 1-pixel disparity represented 10″. Two 5.5Δ based out Risley prisms were placed in front of each eye to assist fusing (Figure 1). The luminance of the smartphone display was measured with a screen luminance meter (SM208, M&A Instrument Inc., Shenzhen, China). A program written with C# was utilized to produce all random dot stereograms with crossed disparity.

### 2.2. Establishing the Parameters of Test Images

#### 2.2.1. Original TNO Stereo Test

**Figure 2:**
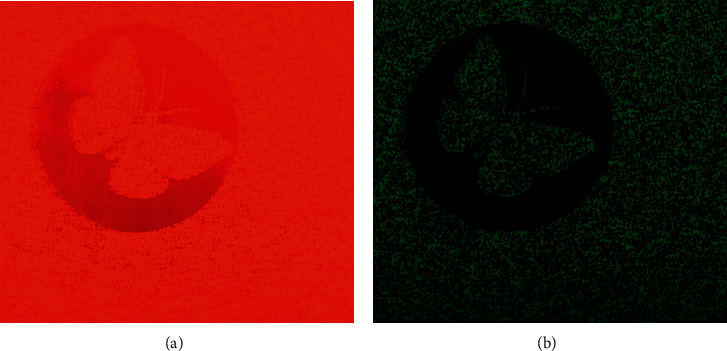
Top left corner of plate I of the TNO stereo test is used to get the picture parameter: (a) mounting red filter in front of a 50 mm lens; (b) mounting green filter in front of a 50 mm lens. The shooting parameter is ISO 800, the aperture value is 2.8, and the shutter speed is 1/200 s.

**Figure 3:**
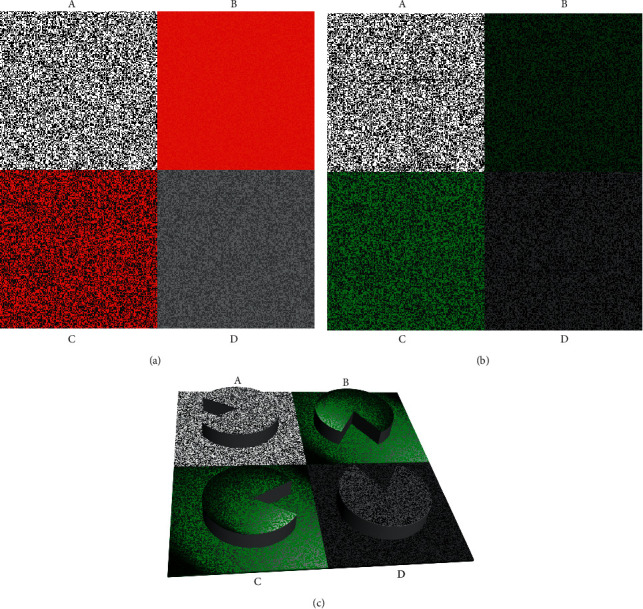
Legend of the test picture I (80″). (a) Seeing by the left eye: A, composed of black dots (0, 0, 0) and white dots (255, 255, 255); B, composed of dark red dots (200, 0, 0) and light red dots (255, 50, 0); C, composed of red dots (230, 0, 0) and gray dots (60, 60, 60); D, composed of dark gray dots (125, 125, 125) and light gray dots (165, 165, 165). (b) Seeing by the right eye: A, composed of black dots (0, 0, 0) and white dots (255, 255, 255); B, composed of dark green dots (0, 20, 0) and light green dots (0, 95, 15); C, composed of green dots (0, 138, 0) and gray dots (60, 60, 60); D, composed of dark gray dots (15, 15, 15) and light gray dots (110, 110, 110). (c) The simulation of the perceptions generated by the test pictures (a) and (b). The missing section of the pie is left, down, right, and up in A, B, C, and D, respectively. The disparity of stereo targets is 80″. The color of B and C may appear reddish, greenish, or flashing depending on the reactions of different people. This simulation imitates a subject who felt the flashing of green light for figure B and C when fusing two images.

To imitate the actual circumstances of a human being watching the TNO stereo test wearing red-green glasses, we set up a test with a camera (Nikon D810, Nikon Corp., Japan) equipped with a 50 mm lens (Carl Zeiss Makro-Planar T^*∗*^ 50 mm F2, Cosina Co., Ltd., Nagano, Japan) to collect data. Plate I, which included butterfly, was adopted as the sampling template. The photograph was taken in our clinic room, with normal outside light transmitted through windows; however, direct sunlight was avoided. A standard gray card was used to calibrate the white balance. The shooting distance was approximately 40 cm. Matrix photometry was also adopted. The International Organization of Standardization (ISO) sensitivity was set at 800, and the aperture value was set at 2.8. Under normal conditions, the shutter speed was set at 1/1000 s to shoot plate I according to the calculation of the camera's measurement system. The red filter of the TNO glasses was removed and fixed beyond the lens of the camera, following which, the shutter speed was decreased to 1/400 s to shoot plate I according to the automatic calculation of the camera. Thereafter, the red filter was removed and changed to the green filter of the TNO glasses. When the green filter was used to shoot plate I, the shutter speed was 1/100 s by automatic calculation. We chose a middle value (1/200 s) to balance the images between the two eyes. The actual photograph is shown in Figure 2. The reddish photograph was overexposed, while the greenish photograph was underexposed. In the reddish photograph, the RGB (red, green, blue) code of a dark red color was (200, 0, 0), and (255, 50, 0) for a light red color. In the greenish photograph, the dark green and light green colors were (0, 20, 0) and (0, 95, 15), respectively. The parameters are summarized in Table 1, and the test pictures are shown in Figures 3(a)-B and 3(b)-B.

#### 2.2.2. Isoluminant Red and Green Pictures

The reddish picture consisted of red (230, 0, 0) dots and grayish (60, 60, 60) dots, and the greenish picture consisted of green (0, 138, 0) dots and grayish (60, 60, 60) dots. The luminances of the red and green colors were all 15 cd/m^2^, and those of the grayish were 5 cd/m^2^. The parameters are summarized in Table 1, and the test pictures are shown in Figures 3(a)-C and 3(b)-C.

#### 2.2.3. Grayscale Pictures

Four pictures were drawn with the RGB code (200, 0, 0), (255, 50, 0), (0, 20, 0), and (0, 95, 15), respectively, and then transmitted to smartphones. For a white picture (255, 255, 255), the luminance of the screen was 126 cd/m^2^. The luminance of dark red, light red, dark green, and light green was 11 cd/m^2^, 26 cd/m^2^, 0.4 cd/m^2^, and 7 cd/m^2^, respectively. The isoluminant grayscale codes of the four colors mentioned above were (125, 125, 125), (165, 165, 165), (15, 15, 15), and (110, 110, 110), respectively. The parameters are summarized in Table 1, and the test pictures are shown in Figures 3(a)-D and 3(b)-D.

#### 2.2.4. Black and White Dot Pictures

The picture consisted of black (0, 0, 0) dots and white (255, 255, 255) dots. The parameters are summarized in Table 1, and the test pictures are shown in Figures 3(a)-A and 3(b)-A.

### 2.3. Participants

A total of 32 participants (10 men and 22 women), aged 20–28 years, were recruited. The best corrected visual acuity was no less than 0 logarithm of the minimum angle of resolution (logMAR) for each eye. The stereoacuity was at least 40″ (evaluated by the Fly Stereo Acuity Test). All participants gave their informed written consent before taking part in the study. The research protocol observed the tenets of the Declaration of Helsinki and was approved by the ethics committee of the Second Hospital of Jilin University (no. 2020-110).

### 2.4. Test Procedure

Eight disparities were utilized to design the test pages, the disparity setting ranged from 8-pixel to 1-pixel. Quantitative measurement in the TNO stereo test was set as one disparity level tested twice. Therefore, each disparity was tested twice in this experiment, and thus 16 pages were designed.

Four squares, including original, isoluminant, grayscale, and black-white images, were closely arranged to assemble in one square. The order of arrangement of the 4 small squares in the large square was randomly selected. The disparity was the same on one test page. The RGB code and luminance are shown in Table 1. The subject was asked to distinguish the direction of the gap of the pie in the sequence from the top left, top right, lower left, and lower right. The test started from 8-pixel page 1, then 8-pixel page 2, 7-pixel page 1, etc. When the subject misdirected at least one image at a certain level, the upper level was recorded as the threshold of him/her. For example, a participant who scored 40″ meant that he/she passed all of the 10 test pages but failed at least one test page in 3-pixel.

### 2.5. Statistical Analyses

PASW Statistics 18.0 (SPSS Inc., Chicago, IL, USA) was used to process the data. The Shapiro–Wilk test was used to explore the distribution of the data. When the data met a normal distribution, parametric tests were carried out; that is, a one-way analysis of variance was used to detect the differences among groups. If the data did not meet normal distribution, nonparametric tests were carried out; that is, the Friedman test was used to detect differences among groups. For the four groups in total, six comparisons were conducted between every two groups. *P* < 0.008 (0.05/6) was used as the threshold for statistical significance (paired *t*-test for parametric tests or Wilcoxon signed-rank test for nonparametric tests).

## 3. Results

None of the data of the four groups met normal distribution (Table 2, the Shapiro–Wilk test, *P* < 0.05). Nonparametric tests were used to analyze the data.

**Figure 4:**
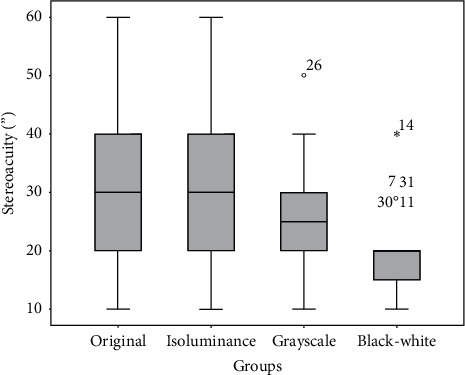
Boxplot of the stereoacuity of the four groups. The line perpendicular to the whisker below the box represents the minimum value; the lower edge of the box represents the first quartile; the thick solid line is the median; the upper edge of the box represents the third quartile; the line perpendicular to the whisker above the box represents the maximum value. Circles and star represent outliers.

The median stereoacuity and interquartile range for the original, isoluminant, grayscale, and black-white groups were 30″ (20″), 30″ (20″), 25″ (10″), and 20″ (7.5″), respectively (Figure 4). A significant difference was found among the four groups (Friedman test, chi-square = 50.985, *P* < 0.001). The Wilcoxon signed-rank test was used to detect differences between the groups: the original group vs. the black-white group, *Z* = −4.378, *P* < 0.001; the original group vs. the isoluminant group, *Z* = −2.828, *P*=0.005; the original group vs. the grayscale group, *Z* = −3.251, *P*=0.001; the isoluminant group vs. the grayscale group, *Z* = −2.140, *P*=0.032. Using the significance level *P* < 0.008 set before the comparisons, the stereoacuity of the original group was significantly worse than that of the isoluminant, grayscale, and black-white groups. However, no significant difference was found between the isoluminant and grayscale groups.

Spearman's rho was performed between the original group and the isoluminant group (correlation coefficient = 0.952, *P* < 0.001). The 95% confidence interval (CI) of the correlation coefficient was 0.901–0.988, calculated using the bootstrap method. The correlation coefficient between the original and grayscale groups was 0.771 (*P*=0.001). The 95% CI of the correlation coefficient was 0.550–0.916. Although the correlation coefficient of the original group and the isoluminant group was higher than that of the original group and the grayscale group, it was difficult to conclude that a significant difference existed between the two coefficients due to the overlapping between two 95% CIs.

## 4. Discussion

As a traditional method to evaluate stereopsis, the TNO stereo test has been used over 40 years. It is easy to operate for an examiner and also easy to understand by the participant being evaluated. However, some studies have reported that the TNO test results may overestimate the value of stereopsis compared with those of other tests [2–10].

The TNO stereo test belongs to a type of global stereopsis test, while many comparison tests conducted belongs to local stereopsis test. Distinguishing random dot-based graphs may be more difficult than that of contour-based graphs. However, this hypothesis was not supported by several studies [14, 15]. The smaller dot size TNO adopted may affect the test result. However, dot size unlikely accounted for the difference in thresholds between the TNO and the other stereo tests in Vancleef's study [14]. The dot size of the TNO was larger than that of many other random dot stereopsis used in the clinic [16]. To avoid the effect of dot size, the smallest dot used in our picture was 6 × 6 pixel, which is equivalent to 1 min arc at 65 cm. The visual acuity of the participants recruited in the experiment was no less than 0 logMAR; thus, the dots in the images could be resolved clearly by all of them.

In our previous study, anaglyph glasses used to separate binocular viewing were one of the reasons for the overestimation of the stereopsis test values in a separate manner. The red-green filters caused two imbalances, chroma and luminance, between the two eyes. Previous studies evaluating the test results tended to adopt commercial stereopsis tests. The minimum threshold may be different, and the test step range may be too large for noticing small differences. We used a computer to create test pictures to imitate the TNO stereo test. Pacman was adopted as a stereo symbol. Four types of test patterns were designed. The first pattern was created to imitate the original TNO stereo test while wearing anaglyph glasses and aimed to maintain the same color and brightness difference as in the actual TNO test. Two 4K smartphones were utilized as test tools. The phone on the left side imitated the condition of the left eye wearing a red filter; the phone on the right side imitated the condition of the right eye wearing a green filter. The second pattern kept the red and green colors but balanced the brightness between two images seen by the left and the right eye. The third pattern eliminated the chromatic factors but maintained the brightness between the two images. The fourth pattern was designed with black and white random dots.

To find a slight difference between tests, the participants were chosen were young students with stereoacuity no less than 40″. All participants completed the test successfully. The results showed that the stereopsis of the original TNO group was significantly worse than that of the isoluminant group (*P*=0.005) and the grayscale group (*P*=0.001). The first comparison balanced the luminance factor, and the second comparison balanced the chromatic factor. The imbalance of luminance between the two eyes is due to wearing TNO anaglyph glasses and affects the test results significantly, and so does the chromatic factor.

The nervous system that processes luminance, chromatic components, and stereoacuity may involve the parvocellular and magnocellular pathways. Three stereopsis mechanisms, first-order luminance stereopsis, second-order luminance stereopsis, and first-order chromatic stereopsis, involve the processing of stereoscopic depth information [1, 17, 18]. The relationship between contrast and stereopsis has been discussed for several decades. The consensus was that the stereopsis thresholds were independent of contrast at relatively high contrasts, while thresholds changed rapidly in low-contrast conditions [19–21]. The contrast significantly affected the stereopsis thresholds under our test environment. The mechanism of the nervous system that processes the information taken from the retina involving chromatic factors to set up stereopsis is complicated. Chromatic information can assist in solving the stereo correspondence problem [17, 22, 23]. Most studies related to chroma and stereopsis have tended to study the same chromatic images transmitted to both eyes to form stereopsis [18, 24–27]. Few have researched the binocular field involved in the effect of a different eye observing the image with a different color. The imbalance chromatic factor significantly affected the stereopsis thresholds in our test.

This conclusion was different from that of Cornforth's study [28]. In the research of Cornforth, the retinal illuminance difference caused by red and green filters did not significantly affect stereopsis. The difference might have been caused by the different stereo test step ranges between the two studies. The red-green glasses may have also been different. In the study by Simons, the transmittance of the green filter was higher than that of the red filter [29]. However, in red-green glasses matching the 19^th^ edition of the TNO stereo test, the transmittance of the red lens was higher than that of the green lens in our study [11]. The edition of the TNO stereo test modified by the manufacturer may lead to clinically important differences [30].

When comparing the test values between the isoluminant and black-white groups, a significant difference was found. A significant difference existed between the grayscale and black-white groups. The second comparison is related to another issue, that is, the relationship between the interocular luminance difference and stereopsis. When the contrast was reduced by the same amount, the stereopsis of binocular changing was better than that of changing only in one eye [31, 32]. This confirmed that the luminance difference between the two eyes was a factor that affected the test result when wearing the TNO anaglyph glasses.

Although the correlation coefficient between the original group and the isoluminant group was higher than that between the original group and the grayscale group, the overlap of the 95% CI implied that the difference was not significant. It was difficult to determine the exact threshold of the degree of deviation of chromatic or luminance that would affect the stereopsis test. However, both the chromatic factor and luminance factor were significant influencing factors that affected the test results under certain conditions, which included wearing the TNO stereo test glasses.

It cannot be asserted that the TNO stereo test is not a suitable stereopsis test. Moreover, the TNO stereo test is an excellent tool for evaluating stereopsis [4]. However, inconformity existed in many of stereo tests and it cannot compare the stereoscopic vision of different cohorts with different tools. Furthermore, it cannot track the changes in stereopsis using different test methods.

A limitation of this study was that the imitation of the test pages may differ from the actual circumstances in which a human watches the test material with the TNO glasses. The test parameter obtained from the smartphone display may vary to a certain extent under different test environments. The participants were young with good stereopsis; therefore, the responses of the other age groups or people with abnormal stereopsis are not known.

## 5. Conclusions

Anaglyph glasses have played an important role in determining the stereoacuity value assessed with the TNO stereo test; however, the results are overestimated when compared with those of other test measurements. The imbalance of chroma and luminance between the two eyes, caused by the anaglyph glasses, was indicated as one of the reasons for stereopsis overestimation.

## Figures and Tables

**Table 1:** Detailed parameters of test pages.

Group	Right side parameter				Left side parameter			
	Dark element		Light element		Dark element		Light element	
	RGB code	Luminance (cd/m^2^)	RGB code	Luminance (cd/m^2^)	RGB code	Luminance (cd/m^2^)	RGB code	Luminance (cd/m^2^)
Original	200, 0, 0	11	255, 50, 0	26	0, 20, 0	0.4	0, 95, 15	7
Isoluminant	60, 60, 60	5	230, 0, 0	15	60, 60, 60	5	0, 138, 0	15
Grayscale	125, 125, 125	11	165, 165, 165	26	15, 15, 15	0.4	110, 110, 110	7
Black-white	0, 0, 0	0.1	255, 255, 255	126	0, 0, 0	0.1	255, 255, 255	126

RGB: red, green, blue.

**Table 2:** Statistical result of the data.

Group	Shapiro–Wilk test		Wilcoxon signed-rank test							
	Statistic	*P*	Versus original		Versus isoluminant		Versus grayscale		Versus black-white	
			*Z*	*P*	*Z*	*P*	*Z*	*P*	*Z*	*P*
Original	0.931	0.042	—	—	−2.828	0.005	−3.251	0.001	−4.378	<0.001
Isoluminant	0.923	0.025	−2.828	0.005	—	—	−2.140	0.032	−4.226	<0.001
Grayscale	0.897	0.005	−3.251	0.001	−2.140	0.032	—	—	−3.999	<0.001
Black-white	0.802	<0.001	−4.378	<0.001	−4.226	<0.001	−3.999	<0.001	—	—

## Data Availability

All the raw data of this article are shown in the supplementary table. The data of personal identity information will not be made available in order to protect the participants' privacy.

## Supplementary Materials

Supplementary Materials Test results of original, isoluminant, grayscale, and black-white groups.
