# Supplementary material for: Evaluating the Mechanism by Which the TNO Stereo Test Overestimates Stereo Thresholds
Source: J Ophthalmol. 2021 Jan 18;2021:6665638. doi: 10.1155/2021/6665638 (PMC7834831; doi:10.1155/2021/6665638)
Supplement: Supplementary Materials — Test results of original, isoluminant, grayscale, and black-white groups. [file 6665638.f1.pdf]

Supplementary table

Test results of participants

ID	age	Original	isoluminance	Grayscale	Black-white
1	28	10	10	10	10
2	24	20	20	20	20
3	24	10	10	10	10
4	28	30	30	20	20
5	24	20	20	20	10
6	25	40	40	30	20
7	27	40	40	40	30
8	27	20	20	30	20
9	25	30	30	20	20
10	24	20	20	20	20
11	24	40	40	30	30
12	24	30	30	20	20
13	25	20	20	20	20
14	25	60	60	40	40
15	25	20	20	20	10
16	25	20	20	20	10
17	22	30	30	30	20
18	22	30	30	30	20
19	22	40	30	30	20
20	22	50	40	20	20
21	22	50	50	30	20
22	22	30	20	20	20
23	22	40	40	30	20
24	22	30	20	20	20
25	22	50	40	40	10
26	22	50	40	50	20
27	27	30	30	30	20
28	25	50	40	40	20
29	24	30	30	20	10
30	20	50	40	40	30
31	20	30	30	40	30
32	25	10	10	10	10
